# Biodegradable Polymers for the Production of Nets for Agricultural Product Packaging

**DOI:** 10.3390/ma14020323

**Published:** 2021-01-09

**Authors:** Francesco Paolo La Mantia, Manuela Ceraulo, Paolo Testa, Marco Morreale

**Affiliations:** 1 Department of Engineering, University of Palermo, Viale delle Scienze, 90128 Palermo, Italy; paltest94@gmail.com; 2 Consorzio INSTM, Via Giusti 9, 50121 Firenze, Italy; manuela.ceraulo@gmail.com; 3 Faculty of Engineering and Architecture, Kore University of Enna, Cittadella Universitaria, 94100 Enna, Italy; marco.morreale@unikore.it

**Keywords:** biodegradable polymer, fruit packaging, fibers, films, elongational flow

## Abstract

It is well known that the need for more environmentally friendly materials concerns, among other fields, the food packaging industry. This regards also, for instance, nets used for agricultural product (e.g., citrus fruits, potatoes) packaging. These nets are typically manufactured by film blowing technique, with subsequent slicing of the films and cold drawing of the obtained strips, made from traditional, non-biodegradable polymer systems. In this work, two biodegradable polymer systems were characterized from rheological, processability, and mechanical points of view, in order to evaluate their suitability to replace polyethylene-based polymer systems typically used for agricultural product net manufacturing. Furthermore, laboratory simulation of the above-mentioned processing operation paths was performed. The results indicated a good potential for biodegradable polymer systems to replace polyethylene-based systems for agricultural product packaging.

## 1. Introduction

Nets for packaging of fruits and vegetables are usually made from blown films of high-density polyethylene (HDPE) or blends of polyethylenes (PEs) cut into small slices, and then cold drawn. To date, the production of polymer films used for this application relies on polyolefins, either neat or blended, and the main mechanical properties of interest in such application are rigidity and elongation at break. Furthermore, a fundamental property for this application is weldability, which cannot be easily obtained by only using HDPE; this issue is overcome by blending it with a low-density polyethylene (LDPE) or metallocene (M-PE), which make the processing much easier. Therefore, in order to find suitable candidates for replacing polyolefins in these applications, the research should be focused on filmability properties, as well as the ability to be efficiently cold drawn and to give rise to slices with a good rigidity and deformability. At the same time, the large use of plastics in the packaging industry has been increasing the environmental concern; therefore, the interest in the use of biodegradable polymers to replace conventional polymers, in order to reduce the environmental impact caused by inappropriate disposal, has grown [1]. Poly(lactic acid) (PLA) is a thermoplastic, biodegradable, and biocompatible polymer that can be produced from renewable resources [2]. PLA is known to have low toughness at room temperature and low melting resistance compared to conventional polymers, with these factors actually hindering its application in large-scale processes, such as industrial blown film extrusion, blowing, and foaming, where stability to the melt is a fundamental factor [3,4]. The melting resistance of PLA must be, therefore, improved in order to widen its processing frame and therefore its field of application. Copolymerization, the addition of plasticizers, and blending with other polymers are some of the main ways to achieve improvements in the properties of PLA [5]. PLA blends with polymers such as poly(butylene-succinate-co-adipate) (PBSA) [6], poly(butylene succinate) (PBS) [7], poly(butyl acrylate) (PBA) [8], and poly(butylene-adipate-co-terephthalate) (PBAT) [9,10] have been studied in order to improve the mechanical properties of PLA, mainly to increase its toughness. In particular, PBAT is a very flexible biodegradable polymer, despite being somewhat underestimated, due to its lower rigidity. Blends of these polymers can give good properties for agricultural and for packaging applications [11,12]. In spite of this, these polymers also have a significant disadvantage—the production cost limits their large-scale application. In order to overcome the issue, biodegradable blends can be prepared with low-cost reinforcements, such as natural fibers and common minerals, thus reducing the final cost and positively influencing the properties of the matrix, especially with regard to tensile strength and Young’s modulus, while deformability may be reduced by increasing the mineral filler content [13]. Thus, nanofillers have also been investigated in place of traditional microfillers. With specific concern to the production of biodegradable fibers, La Mantia et al. [14] studied the effect of cold drawing on the mechanical properties. It is not easy to obtain polymeric fibers with adequate mechanical properties and tailor them to the specific application. The main ways to “tailor” the mechanical properties of a given biodegradable polymer fiber are based on crystallinity and orientation control. However, the crystallinity can be modified only marginally during the processing, while the orientation of the macromolecules can be controlled, both during hot and cold drawing. Although some data are available on the effects of orientation on the main properties [15], only a few papers have addressed the effects of drawing on biodegradable fibers [16]. In general, it can be stated that the orientation leads to a significant increase in the elastic modulus and the tensile strength (with a subsequent decrease in the elongation at break) along the stretching direction, although this applies to semi-crystalline polymers; amorphous polymers and blends can experience a transition from brittle to ductile behavior, induced by orientation [17,18], with a subsequent increase in the elongation at break as the orientation increases. As regards the effect of cold drawing on the properties of polymeric fibers, the information available in the literature is generally focused on traditional non-biodegradable polymers. Cold drawing was found to significantly improve the elastic modulus, tensile strength, and thermomechanical strength of the fibers compared to hot spun fibers. The elastic modulus showed higher rates of increase in biodegradable systems upon increasing the drawing ratio.

Another promising synthetic biopolymer class, designed to replace conventional plastic in numerous applications, is Mater-Bi, a commercial biodegradable polymer based on biodegradable polyesters from renewable production sources. Mater-Bi has found important commercial applications as it shows interesting mechanical properties, thermal stability, processability, and biodegradability [19,20].

**Scheme 1:**
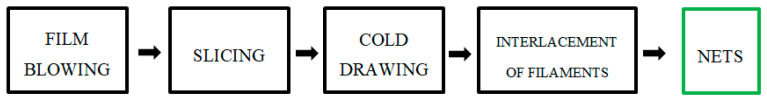
Flow diagram of the net manufacturing process.

In this work, two biodegradable polymer systems were characterized from rheological, processability, and mechanical points of view to evaluate if biodegradable polymers can be suitable for preparing nets for fruits and vegetables, thus replacing traditional, non-biodegradable PE-based nets. As already stated, these nets are prepared by a combined process, where film blowing leads to the production of oriented films, which are then subjected to slicing (or, in other words, films are cut longitudinally in order obtaining numerous, thick slices) and then to cold drawing of the slices, thus obtaining filaments that are directly arranged to form the nets. For the sake of clarity, a flow diagram of the process is shown as Scheme 1. In particular, laboratory simulation was also performed by simulating these two processing operations paths, i.e., film blowing and cold drawing, and by evaluating the obtained results in comparison with the results obtained, under the same experimental procedure, with a typical polymer industrially used for this application. Moreover, the final results were also compared to the mechanical properties of the industrially processed polymer.

## 2. Materials and Methods

### 2.1. Materials and Processing

The materials used in these works were an Ecovio F23B1 sample, i.e., a blend of PBAT with a small amount of PLA and inert, insoluble particles [21] (produced by BASF, Ludwigshafen, Germany); a MaterBi EF51L sample (Novamont, Novara, Italy), i.e., an extrusion grade with proprietary composition, based on biodegradable aliphatic and aliphatic/aromatic polyesters [22]; a HDPE sample commercialized by REPSOL (Madrid, Spain) as Alcudia HDPE R4805D1; and an ethylene-hexene metallocene copolymer (mPE) ENABLE 2005HH from ExxonMobil Chemical (Houston, TX, USA), containing technological adjuvants and thermal stabilizing additives. 

Actually, a blend of HDPE and mPE (80/20) is used for the preparation of the films to produce the nets; therefore, the 80/20 HDPE/mPE blend was considered as the reference material to be compared to the biodegradable polymers. This HDPE/mPE blend was prepared using a co-rotating twin-screw extruder with a length-to diameter ratio (L/D) equal to 35 (OMC, Saronno, Italy), with a 170–180–190–200–210–220–230 °C temperature profile and 220 rpm screw speed. The extruded materials were then pelletized and used for the preparation of specimens to be subjected to mechanical and rheological tests, after cutting them from compression-molded sheets prepared using a Carver (Wabash, IN, USA) laboratory press. Ecovio and MaterBi were always conditioned in vacuum oven before high-temperature processing. 

The film blowing operation on HDPE/mPE, PLA/PBAT (Ecovio), and MaterBi was performed using a Brabender (Duisburg, Germany) equipment, on the basis of a single-screw extruder (D = 19 mm, L/D = 25) and a film blowing unit, operating at the following conditions (Table 1):

Finally, approximately 80 μm thick films were prepared, from which samples for subsequent characterizations were cut off. Furthermore, in order to better simulate, at the laboratory scale, the industrial process, we prepared filaments by cold-drawing of the blown-extruded films (2 mm wide, 5 cm long strips, cut off the blown-extruded films; clamping length was set at 3 cm), which were then subjected to cold (i.e., room temperature) drawing treatment at different draw ratios (DR = 2 and 3) by means of an Instron (Norwood, MA, USA) 3365 universal machine at 10 mm/min deformation speed.

### 2.2. Characterization

Samples were subjected to rheological characterization under shear flow, using both a rotational plate–plate rheometer (Ares G2, TA Instruments, New Castle, DE, USA; dynamic frequency sweep tests at 0.1–100 rad/s frequency range, strain = 5%) and a capillary rheometer (Rheologic 1000, Ceast, Pianezza, Italy; capillary size L = 4 cm, D = 1 mm); non-isothermal elongational flow characterization was performed on the same apparatus by means of a dedicated drawing unit, located at the exit of the capillary die. Melt strength (MS) and breaking stretching ration (BSR) were then calculated, according to the procedures described elsewhere [23].

Mechanical (tensile) characterization on samples coming from the compression molded sheets and blown films and on filaments coming from cold drawing of the blown films was carried out using a Instron (Norwood, MA, USA) mod. 3365 universal machine, with a 3 cm clamping length, a first crosshead speed of 1 mm/min (until 3 min), and then a second crosshead speed increase to 100 mm/min, up to specimen failure.

## 3. Results and Discussion

The extrusion profile and in particular the temperature at the die reported in Table 1 for the conventional polymer is the extrusion profile usually adopted in the industrial extrusion for this material and was adopted in this work. The extrusion temperature profiles for the two biodegradable polymers were chosen on the basis of the flow curves, in particular on the basis of the flow curve at the typical extrusion velocity gradient range in an extruder, which is usually in the range of 100–300 s^−1^.

**Figure 1:**
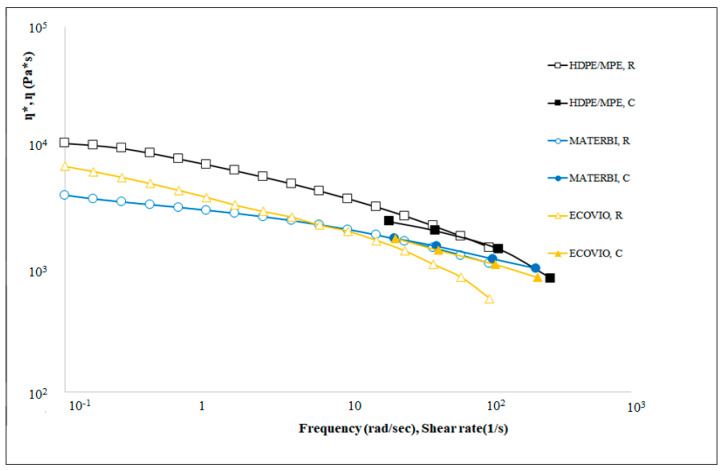
Flow curves of the three polymers at different temperatures. Data were taken both in a rotational rheometer (open points) and in a capillary viscometer (full points).

In Figure 1, the flow curves measured from rheological tests in a rotational rheometer (complex viscosity, η*, vs. frequency) and in a capillary viscometer (viscosity, η, vs. shear rate) for all the materials, at the three different temperatures (230 °C for the conventional polymer blend, 170 °C for the two biodegradable systems), are shown (data reproducibility: ±5%).

As is well evident, the superposition between the curves from the rotational rheometer and the capillary viscometer occurred for the conventional blend and for the MaterBi, but the Cox–Merz rule did not seem to be followed by the Ecovio; this is in agreement with other studies on heterogeneous, multi-phase systems containing solid particles [24].

It can be observed that the highest levels of viscosity were shown by the HDPE/mPE blend and the lowest (on average) by the Ecovio. In the higher shear rate range, the curves tended to approach each other (in particular, with regard to those obtained at the capillary rheometer), and this meant that the extrudability of the three samples was similar.

**Figure 2:**
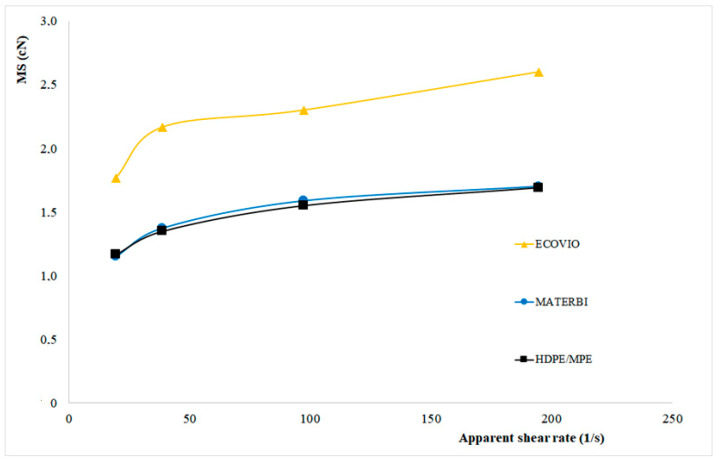
Melt strength of the three polymers as a function of the apparent shear rate. Temperatures and symbols are as in Figure 1.

**Figure 3:**
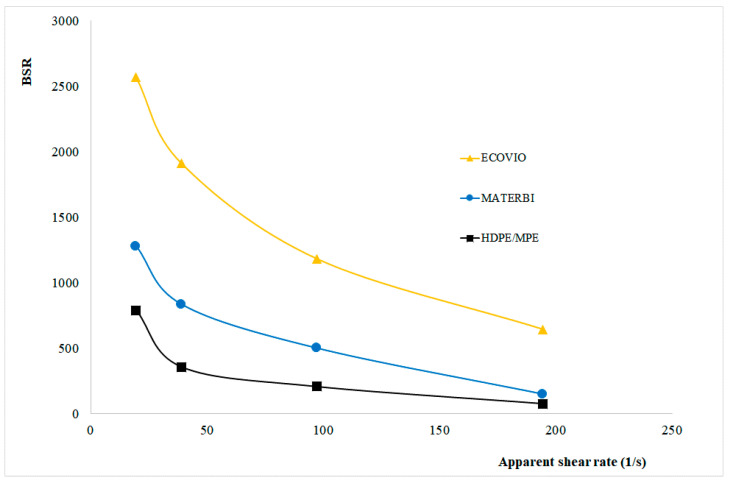
Breaking stretching ratio of the three polymers as a function of the apparent shear rate. Temperatures and symbols are as in Figure 1.

The filmability of the polymers in a typical film blowing process is controlled by the behavior under non-isothermal elongational flow. For this reason, the melt strength (MS) and breaking stretching ratio (BSR) were measured at the same temperatures of the rheological tests, which is reported in Figure 2 and Figure 3 (data reproducibility: ±7%).

The BSR values showed that the conventional polymer system was the less stretchable melt, while the two biodegradable systems presented higher values. The combined results of MS and BSR clearly gave evidence that also the filmability of these two biodegradable polymer systems was at least similar or even better than that of the conventional polymer blend.

The results from the tensile tests, elastic modulus (E), tensile strength (TS), and elongation at break (EB), measured on isotropic (i.e., compression molded) specimens, are shown in Table 2 below.

Both of the biodegradable systems showed lower rigidity (on the basis of the elastic modulus values) than the HDPE/mPE blend, while they can be considered competitive in terms of ductility; with regard to the tensile strength, the MaterBi sample was even better than the HDPE/mPE blend, while Ecovio’s values were lower.

The same mechanical properties on the anisotropic samples (blown films) are shown in Table 3 for MD and TD specimens.

According to the experimental results, the films were oriented in both directions at different levels as the elastic modulus and the tensile strength were higher than the same values of the isotropic sheet and the elongation at break was lower. Due to the processing conditions, the orientation was more relevant in the machine direction.

With regard to the elastic modulus in MD, there were significant differences between the two biodegradable systems and the non-biodegradable system. On the other hand, the tensile strength (in both directions) was competitive or even higher; MaterBi showed higher values than Ecovio. As for the elongation at break, the biodegradable systems showed lower values than the conventional material. These comments can be made also with concern to the TD direction. It is worth mentioning, however, that the following drawing action, meant to produce the final filaments for the net manufacturing, was performed on the strips along the machine direction.

Table 4 reports the mechanical properties of Ecovio and MaterBi cold-drawn filaments at increasing draw ratios, compared to the same properties measured for a standard filament, cut from a commercial net for fruit packaging. First, it can be observed that the filaments of both of the two biodegradable materials strongly improved their stiffness and tensile strength upon increasing the draw ratio; on the other hand, the elongation at break decreased. All of these results were in agreement with the expectations, since the cold drawing increased the orientation degree of the macromolecules and reduced their reciprocal slipping ability. However, the elongation still kept values higher than that of the standard filament and, at the same time, the improvement of elastic modulus and tensile strength allowed us to reduce the gap with the standard filaments. Moreover, the values of the elastic modulus of Mater-Bi, at this draw ratio, became fairly similar to the conventional filament.

**Figure 4:**
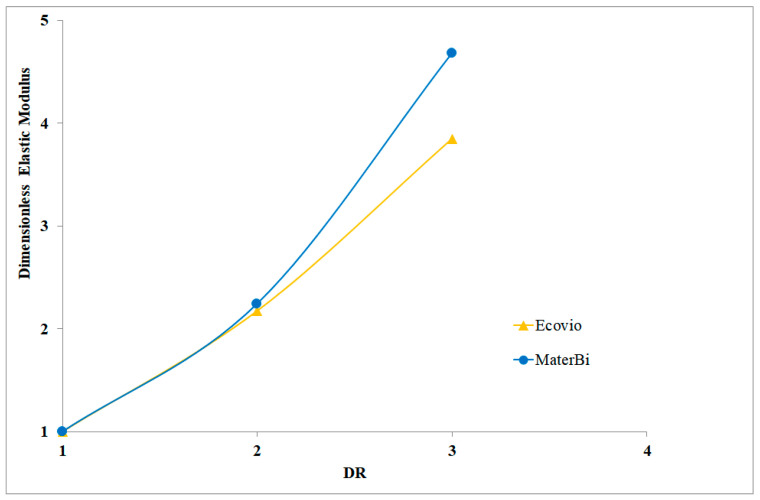
Dimensionless elastic modulus as a function of the draw ratio.

**Figure 5:**
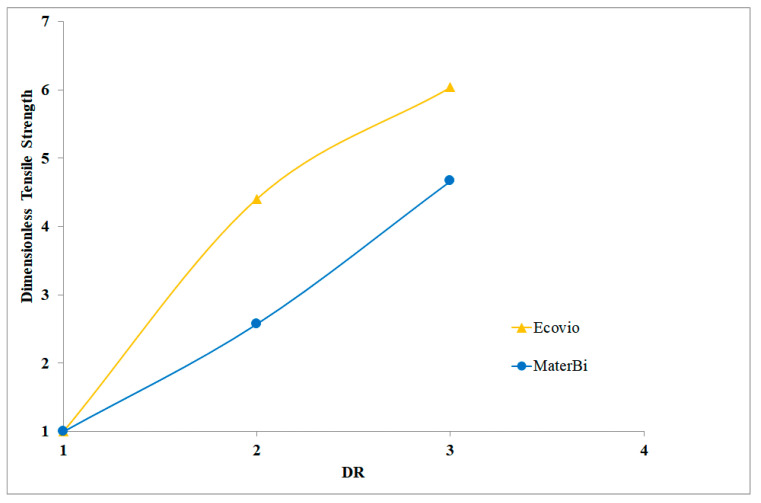
Dimensionless tensile strength as a function of the draw ratio.

**Figure 6:**
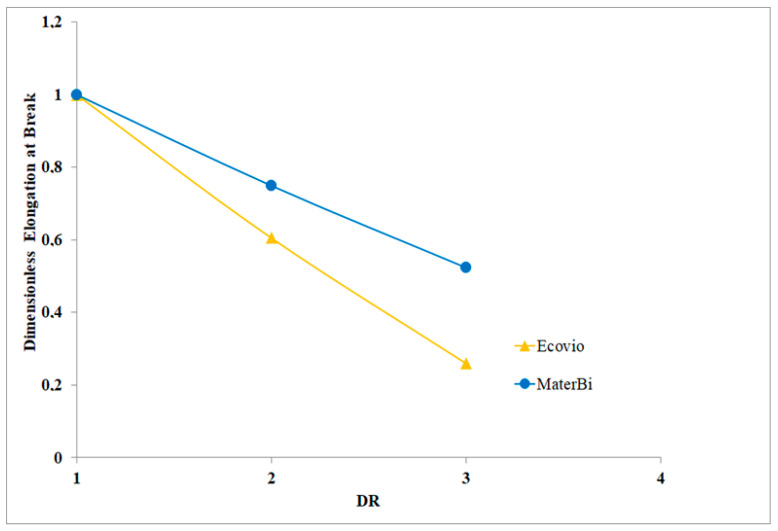
Dimensionless elongation at break as a function of the draw ratio.

In order to better assess the actual effect of increasing the draw ratio, and thus the orientation degree, of cold-drawn filaments, we calculated and plotted the dimensionless tensile properties (i.e., the ratio between the actual average value of that property and the average value of the same property measured at DR = 1, which is that of the corresponding strip obtained from the blown film), as shown in Figure 4, Figure 5 and Figure 6. It can be observed that the mechanical properties at DR = 1 were slightly higher in the MaterBi sample. The sole effect of cold drawing the filament significantly improved the properties of both; moreover, it appeared that, at higher DRs, the orientation was slightly more efficient in the MaterBi sample. As regards EB, as already stated, this decreased upon increasing the orientation, but the decrease was proportionally lower in the MaterBi. The overall results allowed us to state that the orientation significantly improved the mechanical properties with primary importance for fruit net application, i.e., modulus and tensile strength; moreover, the reduction in the elongation at break (and thus, deformability) is a beneficial result in this context.

## 4. Conclusions

In this work, two biodegradable polymer systems were compared to a traditional, non-biodegradable polyethylene-based blend, in view of possible application for the production of nets for the food packaging industry, particularly for the packaging of fruits and vegetables.

The industrial process was accurately simulated on the laboratory scale via film blowing, cutting of the film into thin strips and cold drawing to obtain drawn filaments. The investigated systems were subjected to rheological and mechanical characterization.

From the rheological characterization, we assessed that the processability of the biodegradable systems was, on average, comparable to that of the traditional, non-biodegradable blend; the same applied to the non-isothermal elongational flow parameters (related to filmability).

Mechanical characterization on compression-molded samples showed that MaterBi’s rigidity was significantly lower than that of HDPE/mPE, while the tensile strength was slightly higher. Tests on the MD films showed that both of the biodegradable polymers (and, in particular, the MaterBi sample) can be considered as alternatives for the reference non–biodegradable films.

The tests on the drawn filaments demonstrated that the elastic modulus and the tensile strength can be dramatically improved by increasing the draw ratio.

## Figures and Tables

**Table 1:** Investigated systems and processing conditions.

Polymer System	Temperature Profile (°C)	Screw Speed (rpm)	Draw Ratio, DR	Blow-Up Ratio, BUR
HDPE/mPE	170–190–210–230–230–230	100	3	2
MaterBi	120–130–140–150–160–170	80	3	2
Ecovio	150–160–170–170–170–170	80	3	2

**Table 2:** Tensile properties of isotropic, compression-molded samples.

Sample	E (MPa)	TS (MPa)	EB (%)
HDPE/mPE	285 ± 5	17.2 ± 0.3	743 ± 10
MaterBi	110 ± 7	21.5 ± 0.6	617 ± 19
Ecovio	98 ± 1.5	9.2 ± 0.5	633 ± 14

**Table 3:** Tensile properties in machine direction (MD) and transverse direction (TD) of the anisotropic (film) samples.

Sample	MD			TD		
	E (MPa)	TS (MPa)	EB (%)	E (MPa)	TS (MPa)	EB (%)
HDPE/mPE	393 ± 8	24.3 ± 0.7	480 ± 17	293 ± 6	19.1 ± 0.6	583 ± 20
Ecovio	212 ± 7	27.1 ± 1	385 ± 20	119 ± 6	17.3 ± 0.8	495 ± 23
MaterBi	327 ± 8	33 ± 2	319 ± 19	269 ± 6	27.7 ± 1.8	469 ± 21

**Table 4:** Tensile properties of the biodegradable polymer filaments, at increasing draw ratios, compared to the reference, industrially manufactured, non-biodegradable polymer filament.

System	DR	E (MPa)	TS (MPa)	EB (%)
Ecovio	1	212 ± 7	27.1 ± 1	385 ± 20
	2	461 ± 20	119 ± 4	233 ± 19
	3	816 ± 13	163 ± 3	100 ± 29
MaterBi	1	327 ± 8	33 ± 2	319 ± 19
	2	734 ± 29	85 ± 6	239 ± 16
	3	1531 ± 37	154 ± 5	167 ± 17
Reference industrially manufactured filament	–	1904 ± 100	176 ± 10	89 ± 9

## Data Availability

No new data were created or analyzed in this study. Data sharing is not applicable to this article.
